# Surveillance for probable COVID-19 using structured data in the electronic medical record

**DOI:** 10.1017/ice.2020.359

**Published:** 2020-07-23

**Authors:** Patrick C. Burke, Rachel Benish Shirley, Matthew Faiman, Eric W. Boose, Robert W. Jones, Amy Merlino, Steven M. Gordon, Thomas G. Fraser

**Affiliations:** 1 Department of Infection Prevention, Cleveland Clinic, Cleveland, Ohio; 2 Enterprise Quality, Cleveland Clinic, Cleveland, Ohio; 3 Cleveland Clinic Community Care, Cleveland Clinic, Cleveland, Ohio; 4 Information Technology, Cleveland Clinic, Cleveland, Ohio; 5 Department of Infectious Disease, Cleveland Clinic, Cleveland, Ohio

*To the Editor—*Because of limited testing for COVID-19 in the community, on April 8, 2020, the Ohio Department of Health adopted the Council for State and Territorial Epidemiologists’ (CSTE) case definition for COVID-19,^1^ requiring confirmed and probable cases of the disease to be reported to public health authorities within 24 hours of identification. Probable COVID-19 includes patients with compatible clinical and epidemiologic characteristics for whom no confirmatory laboratory testing has been performed and no alternative more likely diagnosis is made. Eliminating requisite laboratory confirmation for reporting cases of COVID-19 to the public health department presents a unique challenge for healthcare systems that traditionally rely on laboratory-based notification of reportable infectious diseases.

At the same time, the Cleveland Clinic was optimizing the use of our virtual encounter platforms to minimize the risk of severe acute respiratory coronavirus virus 2 (SARS-CoV-2) exposure in the traditional healthcare setting according to guidance from the Centers for Disease Control and Prevention. Virtual visits at the Cleveland Clinic are synchronous live video- and audio-enabled encounters that take place through our Express Care Online format or other commercial apps, eg, Apple FaceTime. In 2018, the Cleveland Clinic provided 33,789 virtual visits through Express Care Online. We saw the increasing demand for virtual visits in the wake of the COVID-19 pandemic as an opportunity to leverage documentation in the medical record for probable COVID-19 surveillance and reporting.

## Methods

We developed a standardized clinical note template in our electronic medical record (EPIC, Epic Systems, Verona, WI) with COVID-19 Smart Data Elements (SDEs) aligned with the clinical and epidemiologic criteria in the CSTE case definition. Patient responses to these ‘yes’ or ‘no’ fields guide the healthcare provider in diagnostic and testing decision making (Supplemental Material online). Virtual visit providers at the Cleveland Clinic are prompted to use this note template when the provider selects “COVID-19 concern” as the patient’s chief complaint. Patients could be referred for SARS-CoV-2 testing if they were in Ohio, had a high-risk chronic condition or age, and had at least 2 of the following symptoms: fever, cough, shortness of breath, myalgia, diarrhea, anosmia, or loss of taste. SDEs from virtual visits in the structured format enable rapid daily extraction from our Enterprise Data Vault (EDV) and reporting to public health within 24 hours.

We queried the EDV for patients seen by virtual visit with at least 1 major COVID-19 criteria (ie, cough, shortness of breath, or difficulty breathing) or at least 2 minor COVID-19 criteria (ie, measured or subjective fever, chills, myalgia, headache, sore throat, new olfactory and/or taste disorder). Rigors, a minor criterion in the CSTE definition, was not included as a discrete field in our note template. We used the presence of any of the following viral infection–related *International Classification of Disease, Tenth Revision* (ICD-10) codes as our indication that no alternative diagnosis had been made: B34.9, viral illness; R68.89, suspected COVID-19 virus infection; Z20.828, exposure to COVID-19 virus; Z71.89, educated about COVID-19 virus infection; B97.29, other coronavirus as the cause of diseases classified elsewhere; or J22, lower respiratory infection (eg, bronchitis, pneumonia, pneumonitis, pulmonitis). We considered patient residence in the United States to satisfy the epidemiologic linkage criteria of the probable case definition, “residence in an area with sustained, ongoing community transmission of SARS-CoV-2.”^2^ We included in our query whether the patient reported close contact with a confirmed or probable case of COVID-19.

## Results

Between April 15 and April 21, 2020, 526 patients from 12 states were seen by virtual visit for COVID-19 concerns; 218 (41%) of these met the CSTE case definition for probable COVID-19 and were reported to the public health department. Also, 167 patients who otherwise met the definition for probable COVID-19 were subsequently tested for SARS-CoV-2, so were not reported as such; 16 (10%) tested positive. Of the 35 patients tested that did not meet the probable case definition due to a lack of clinical criteria or the presence of an alternative more likely diagnosis, 5 (14%) tested positive. During the same week, the Cleveland Clinic reported 353 cases of laboratory-confirmed COVID-19 diagnosed in our hospital, emergency departments, and our drive-through testing center.

Overall, 171 patients (78%) with probable COVID-19 reported at least 1 major clinical criteria, whereas 47 patients (22%) reported no major criteria but 2 or more minor criteria. Furthermore, 139 patients (64%) were classified as probable COVID-19 cases based on residence or travel in the United States, without known close contact with a confirmed or probable case of COVID-19. The clinical and epidemiologic characteristics of patients seen via virtual visits are shown in Table 1.

**Table 1.** Clinical and Epidemiologic Characteristics of Patients Seen at the Cleveland Clinic Virtual Visit for COVID-19 Concern Who Did and Did Not Meet the CSTE Case Definition for Probable COVID-19, April 15–April 21, 2020

Characteristic	Probable COVID-19 (N = 218), No. (%)	Not Probable COVID-19 (N = 106), No. (%)^a^
**Major clinical criteria**		
Cough	160 (74)	34 (32)
Shortness of breath	80 (37)	8 (8)
Difficulty breathing	39 (18)	5 (5)
**Minor clinical criteria**		
Fever (measured or subjective)	87 (40)	14 (13)
Chills	120 (55)	11 (10)
Rigors^b^	…	…
Myalgia	114 (52)	17 (16)
Headache	119 (55)	30 (28)
Sore throat	96 (44)	27 (25)
New olfactory and taste disorder(s)	37 (17)	2 (2)
**Epidemiologic Link**		
Travel to or residence in an area with sustained, ongoing community transmission of SARS-CoV-2	218 (100)	106 (100)
Close contact with confirmed or probable COVID-19	79 (36)	17 (16)

a Patients that did not meet the probable COVID-19 case definition and no SARS-CoV-2 test was performed before April 26, 2020.

b Symptom not included in COVID-19 Smart Data Elements.

## Discussion

Implementing structured documentation in the electronic medical record for the clinical and epidemiologic characteristics of the CSTE case definition for COVID-19 enabled us to easily find and report additional cases to public health authorities for investigation. Finding these previously undetected cases increased our COVID-19 reports to the public health department by 62%.

The public health value added by surveillance and investigation of probable COVID-19 will become less clear as testing availability increases. Illustrating this point is our finding that, among the patients who would have been probable COVID-19 cases without being tested, fewer than 10% tested positive for SARS-CoV-2. Our findings highlight the need for more widespread testing for SARS-CoV-2 to appropriately allocate scare public health resources for COVID-19 investigation, isolation, and contact tracing.

## Supplementary material

For supplementary material accompanying this paper visit https://doi.org/10.1017/ice.2020.359. click here to view supplementary material
