# Supplementary figures and images for: Surveillance for probable COVID-19 using structured data in the electronic medical record
Source: Infect Control Hosp Epidemiol. 2020 Jul 23:1–2. doi: 10.1017/ice.2020.359 (PMC7419178; doi:10.1017/ice.2020.359)

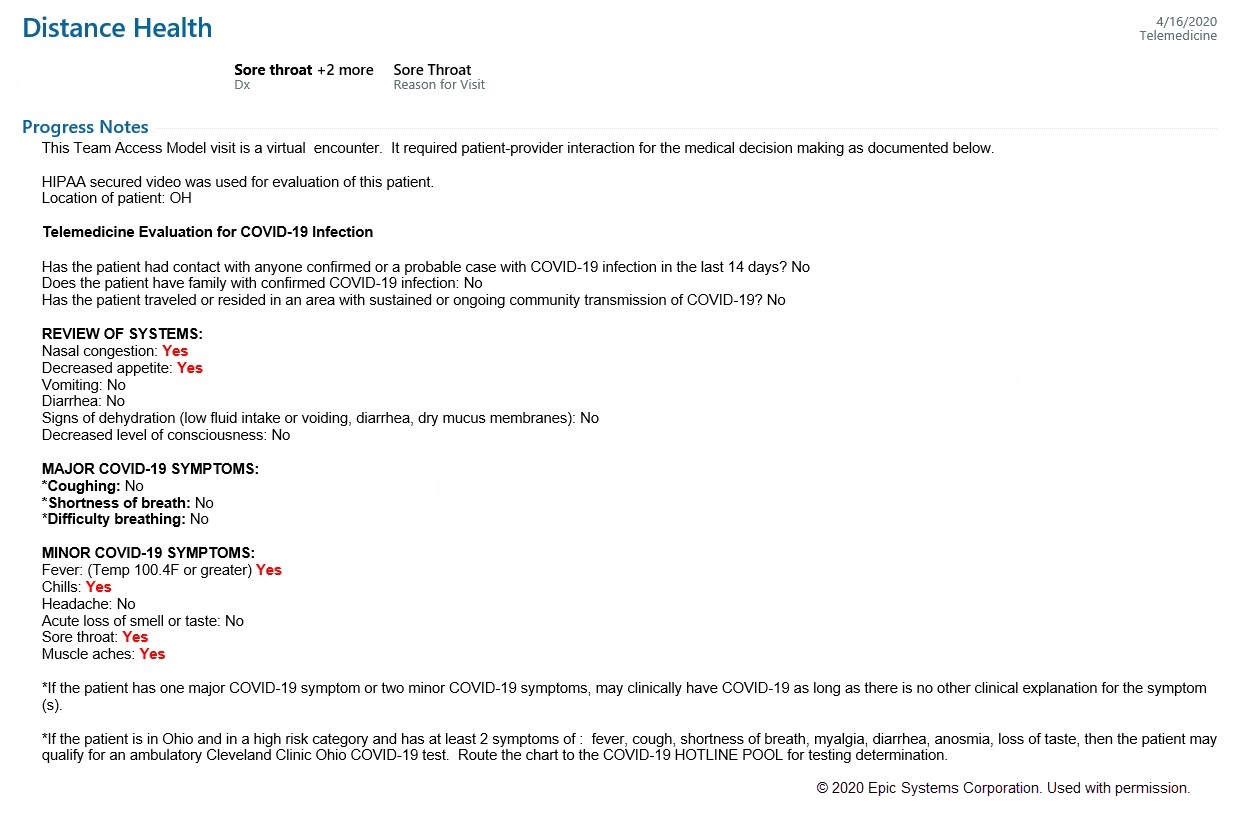

Supplement: Supplementary file 1 [file S0899823X20003591sup001.png]
